# Differentiating Immune Checkpoint Inhibitor-Related Pneumonitis from COVID-19 Pneumonia Using a CT-based Radiomics Nomogram

**DOI:** 10.2174/0115734056399950251003114150

**Published:** 2025-10-21

**Authors:** Fengfeng Yang, Zhengyang Li, Di Yin, Yang Jing, Yang Zhao

**Affiliations:** 1 Department of Radiology, The Second Hospital of Tianjin Medical University, Tianjin 300211, China; 2 Huiying Medical Technology Co., Ltd., Beijing 100192, China

**Keywords:** Radiomics, COVID-19, Chest CT, Immune checkpoint inhibitor therapy, Support Vector Machine

## Abstract

**Introduction:**

We developed and validated a novel CT-based radiomics nomogram aimed at improving the differentiation between checkpoint inhibitor-related pneumonitis (CIP) and COVID-19 pneumonia, addressing the persistent clinical uncertainty in pneumonia diagnosis.

**Methods:**

A total of 97 patients were enrolled. CT image segmentation was performed, extracting 1,688 radiomics features. Feature selection was conducted using variance thresholding, the least absolute shrinkage and selection operator (LASSO) regression, and the Select K Best methods, resulting in the identification of 33 optimal features. Several classification models (K-Nearest Neighbors [KNN], Support Vector Machine [SVM], and Stochastic Gradient Descent [SGD]) were trained and validated using a 70:30 split and fivefold cross-validation. A radiomics nomogram was subsequently developed, incorporating the radiomics signature (Rad-score) alongside clinical factors. It was assessed based on area under the curve (AUC), sensitivity, specificity, and decision curve analysis (DCA).

**Results:**

The SVM classifier exhibited the highest performance, achieving an AUC of 0.988 in the training cohort and 0.945 in the validation cohort. The constructed radiomics nomogram demonstrated a markedly improved predictive accuracy compared to the clinical model alone (AUC: 0.853 vs. 0.810 in training; 0.932 vs. 0.924 in validation). Calibration curves indicated a strong alignment of the model with observed outcomes, while DCA confirmed a greater net benefit across various threshold probabilities.

**Discussion:**

A radiomics nomogram integrated with radiomics signatures, demographics, and CT findings facilitates CIP differentiation from COVID-19, improving diagnostic efficacy.

**Conclusion:**

Radiomics acts as a potential modality to supplement conventional medical examinations.

## 1 INTRODUCTION

In recent years, Immune Checkpoint Inhibitors (ICIs), including programmed cell death protein-1 (PD-1) and its corresponding ligand, programmed death-ligand 1 (PD-L1), have profoundly transformed the landscape of cancer therapy, marking important progress in their clinical use [1]. Immune checkpoint inhibitors function by reinstating the immune system's ability to recognize and combat tumor cells effectively [2]. However, these may also result in a range of immune-related side effects. One such rare but serious side effect is checkpoint inhibitor-related pneumonitis (CIP) [3]. With the growing use of ICIs in cancer therapy, the occurrence of CIP is anticipated to rise even more in the foreseeable future [4]. The lack of specific clinical symptoms in affected patients means chest computed tomography (CT) scans are vital for diagnosing CIP [5].

Severe acute respiratory syndrome coronavirus 2 (SARS-CoV-2) represents a newly identified coronavirus responsible for a highly transmissible respiratory illness known as the 2019 coronavirus disease (COVID-19) [6]. The worldwide dissemination of COVID-19 has led to widespread infections, severe cases, and fatalities across all countries. Consequently, chest CT scans have become essential for screening and assessing the severity of COVID-19. Notably, the clinical manifestations and imaging features of COVID-19 exhibit notable similarities with those observed in CIP, such as nonproductive cough, unresolved dyspnea, and nonspecific interstitial pneumonia [7]. Chest CT findings of COVID-19 pneumonia and checkpoint-inhibitor pneumonitis (CIP) overlap substantially: both frequently present with bilateral, peripheral, and basal-predominant ground-glass opacities (GGO) with or without reticulation, and both may evolve to consolidation or a crazy-paving pattern [8]. Importantly, pooled data indicate that combining initial chest CT with RT-PCR increases sensitivity for COVID-19 diagnosis from 60–70% (RT-PCR alone) to 88–97%, especially during the first 5 days of symptoms, when viral loads may still be below the PCR detection threshold [9]. These similarities pose challenges for healthcare professionals in distinguishing between the two conditions. Accurately differentiating between COVID-19 and CIP is crucial for clinical management, including the treatment of pneumonia and the decision to continue or restart immunotherapy [10].

Radiomics, an emerging field that combines artificial intelligence with medical imaging, provides insights into disease pathophysiology by analyzing quantitative imaging features, thereby revealing information on pathogenesis and etiology [11]. It has been applied to differentiate between benign and malignant tumors [12], predict patient prognosis [13], monitor treatment response [14], and assess gene expres-sion [15]. Research demonstrates that chest CT radiomics can effectively differentiate between immune checkpoint inhibitor-associated pneumonia and radiation pneumonitis in patients with non-small cell lung cancer [16]. Models that combine radiomic features with clinical data have demonstrated enhan-ced discrimination between COVID-19 and non-COVID-19 pneumonia cases [17, 18]. Despite its potential, limited research has been conducted on using radiomics to distinguish between CIP and COVID-19. Exploring the capability of CT radiomics in this context is both critical and promising.

Our research objective is to construct a model for the differential diagnosis of CIP and COVID-19 by employing radiomic characteristics from chest CT in conjunction with clinical or imaging factors.

## 2 MATERIAL AND METHODS

### 2.1 Study Population

The research conducted was a retrospective observational analysis that included individuals diagnosed with bladder cancer who received ICI therapy and developed pneumonitis between July 2020 and June 2023. The research was conducted in accordance with the Declaration of Helsinki, as amended in 2013, and was approved by the Medical Ethics Committee of the Second Hospital of Tianjin Medical University. For this retrospective study, the requirement for informed consent was exempted. Fig. (**1a-e**) illustrates the study protocol in detail.

Each participant underwent a chest CT scan and was selected consecutively from our electronic records. The study included two groups of patients, which were categorized based on their medical history and the findings from the CT imaging, as outlined in the radiological reports. The criteria for inclusion in each cohort were established as follows:

Checkpoint inhibitor-related pneumonitis: This condition involves a group of oncological patients who developed pneumonitis after receiving checkpoint inhibitor therapy. Chest CT scans can exhibit a range of radiographic findings, which may present patterns similar to those observed in cryptogenic organizing pneumonia, acute interstitial pneumonia, and acute respiratory distress syndrome. The identification of pneumonitis induced by ICI therapy primarily relies on clinical assessment. This process involves ruling out other known causes of pneumonia, whether they are microbiological or pharmacological. A key indicator of ICI-induced pneumonitis is the noticeable improvement in symptoms after stopping the medication, along with the effectiveness of medical treatments, especially corticosteroids. Confirmation of the diagnosis is further supported by the resolution of any radiographic abnormalities seen in follow-up chest CT scans [10, 19].

COVID-19: This group consists of consecutive patients showing symptoms such as fever > 37.5°C, dyspnea, cough, and fatigue, who were confirmed to have COVID-19 pneumonia. Confirmation was established through positive reverse transcription–polymerase chain reaction tests on nasopharyngeal or oropharyngeal swabs, as well as positive chest CT scans, with data collected between July 2021 and June 2022 [20].

Exclusion criteria for the study include poor-quality CT images unsuitable for analysis and the presence of other chest conditions such as tuberculosis, chronic obstructive pulmonary disease, and lung cancer on chest CT. Additionally, other forms of pneumonia, including bacterial pneumonia, radiation pneumonia, and non-COVID-19 pneumonia, should also be ruled out. Bacterial pneumonia: Meet any of: a) Procalcitonin ≥0.25 ng/mL on presentation; b) Negative multiplex PCR for typical/atypical respiratory bacteria in nasopharyngeal/sputum sample within 24h of admission; c) For radiographic consolidation: no response to empiric antibiotics within 48h OR negative pre-antibiotic blood/pleural fluid culture. Non-COVID viral pneumonia: Negative multiplex respiratory viral PCR within 24h of admission; OR (if PCR unavailable) two negative rapid antigen tests for influenza A/B and RSV taken 12-24h apart. Radiation pneumonitis: a) No thoracic radiotherapy within the past 12 weeks; b) High-resolution CT pattern inconsistent with organizing pneumonia or radiation recall.

### 2.2 CT Acquisition

The Siemens SOMATOM Definition Edge (Siemens Healthineers, Germany) and GE LightSpeed VCT spiral CT scanners (GE Healthcare, USA) were used for imaging. Patients were positioned supine, and scans were taken at full inspiration using standard doses. The scanning range encompassed the area from the apex of the lung to the costophrenic angle, employing a slice thickness of 5 mm, a tube voltage of 120 kV, and a tube current of 100 mA. The images were generated through a medium-sharp reconstruction algorithm, resulting in a final thickness of 1.25 mm.

### 2.3 Image Segmentation

The initial digital imaging and communications in medicine images were uploaded to the Radcloud platform (Huiying Medical Technology Co., Ltd) for preliminary processing. This step normalized the data to reduce variability from different scanning techniques and improved reproducibility [21]. The CT images were manually delineated by two seasoned radiologists, each possessing more than a decade of expertise in lung CT interpretation, without any prior knowledge of the patients' clinical histories. Subsequently, a senior radiologist conducted a thorough examination of all identified regions of interest (ROIs), who made the final decision on any discrepancies greater than 5% in the delineation of pneumonitis borders [22]. The methodology of the radiomics approach is depicted in Fig. (**1a-e**).

### 2.4 Feature Extraction

Subsequent to defining the volume of interest corresponding to each lesion, A total of 1,688 quantitative imaging features were extracted from CT images utilizing the Radcloud platform (http://radcloud.cn/). These features were systematically organized into three distinct categories. The first group, termed first-order statistics, comprised 126 descriptors that quantitatively depict the distribution of voxel intensities in the CT images through fundamental and commonly used metrics. The second group, which focused on shape- and size-related characteristics, included 14 three-dimensional features that pertain to the geometric attributes of the specified region. The third group, identified as texture features, contained 525 features derived from grey-level run-length and grey-level co-occurrence matrices, which quantify variations in regional heterogeneity. The texture properties emphasize the recurrent local structures present in the images and their underlying organizational patterns. This analytical framework includes 75 features derived from three principal matrices: the Gray Level Co-occurrence Matrix (GLCM), the Gray Level Run Length Matrix (GLRLM), and the Gray Level Size Zone Matrix (GLSZM). Furthermore, texture representation was achieved across multiple resolutions by applying 14 distinct filters. These filters incorporate a variety of techniques, such as index, logarithm, gradient, square value, square root, and the two-dimensional local binary pattern (lbp-2D). Additionally, a variety of wavelet transformations were employed, specifically Wavelet-LHL, Wavelet-LHH, Wavelet-HLL, Wavelet-LLH, Wavelet-HLH, Wavelet-HHH, Wavelet-HHL, and Wavelet-LLL, to facilitate a more thorough investigation of texture.

To assess the reliability of manual segmentation conducted by two radiologists, CT scans from ten randomly selected patients were analyzed by both professionals in a double-blind manner. The consistency of each feature was evaluated utilizing the intra-class correlation (ICC) metric, which was applied both internally and across different observers. Features that demonstrated inadequate reproducibility were removed from further analyses. Specifically, any feature with an ICC value below 0.75 was excluded from further consideration.

### 2.5 Feature Selection

To mitigate the risk of overfitting the constructed signature, a dimensionality reduction of features was performed prior to its development. Radiomic features exhibiting inter- and intra-observer intraclass correlation coefficients exceeding 0.75, along with those demonstrating statistically significant differences between groups, as assessed by one-way analysis of variance (ANOVA), were identified through the Select K Best method and the least absolute shrinkage and selection operator (LASSO) regression model. This selection process was designed to pinpoint the most informative features within the training dataset. Furthermore, the variance threshold method applied a cutoff of 0.8, effectively eliminating features with variance eigenvalues that fell below this threshold. The Select K Best method, recognized as an univariate feature selection approach, utilized p-values to evaluate the association between features and classification outcomes, including all features with p-values lower than 0.05. The LASSO model was configured to incorporate a cross-validation error value of 5 and a maximum iteration limit of 1,000. Subsequently, the selected features were utilized to formulate a radiomic signature, adhering to parameter settings consistent with those established in earlier research [23, 24].

### 2.6 Classifier Training

Based on the clinical data and subsequent imaging assessments, the datasets for validation and training were randomly divided in a ratio of 3:7, using a random seed of 958. Three classification algorithms-k-nearest neighbor (KNN), support vector machine (SVM), and stochastic gradient descent (SGD)-were employed and evaluated through a fivefold cross-validation approach. Five-fold cross-validation was applied to the training set to optimize hyperparameters (*e.g*., SVM kernel, LASSOλ), reducing overfitting. This approach segments the dataset into five distinct portions, sequentially training the model on each segment, and subsequently assesses the algorithm's accuracy by computing the average outcomes across five training iterations. The most effective model for differentiating between CIP and COVID-19 was identified. The efficacy of the feature classifier was further verified and assessed using various metrics, including the area under the curve (AUC), sensitivity, specificity, accuracy, recall, and F1-score, as represented by the receiver operating characteristic (ROC) curve.

### 2.7 Development of a Radiomics Nomogram and Performance Assessment

After selecting key features, a radiomics signature, referred to as the Rad-score, was developed through a linear combination of chosen features along with their associated coefficients obtained from the LASSO method. Additionally, nomograms were developed to determine whether clinical or radiological parameters could further differentiate subtypes of pneumonitis by integrating the Rad-score with clinical variables, rather than relying solely on clinical factors. A calibration plot was employed to evaluate the calibration and the goodness-of-fit of the nomogram. To quantify the predictive performance of both the clinical factors model and the radiomics nomogram regarding prognosis, we computed the C-index for both the training and validation cohorts. Additionally, decision curve analysis (DCA) was performed on the training set to determine the net benefits across various threshold probabilities.

### 2.8 Statistical Analysis

Statistical analyses were conducted utilizing SPSS (version 25.0, IBM) and R statistical software (version 3.3.3; https://www.r-project.org). A univariate analysis was employed to evaluate clinical variables across groups, applying the chi-square test or Fisher's exact test for categorical data, and the Mann–Whitney U test for continuous variables, as appropriate. A one-way ANOVA was used to assess the efficacy of each radiomics feature in distinguishing between CIP and COVID-19. The comparison of the ROC curves from the two datasets was executed using the DeLong test. The predictive performance of the models was evaluated using the validation set, based on thresholds established during the training set. ROC curves were generated using the “pROC” package, while the nomogram was constructed with the “rms” package. Additionally, DCA was carried out using the “dca.R” package. A two-tailed p-value of less than 0.05 was deemed statistically significant.

## 3 RESULTS

### 3.1 Study Population

The investigation comprised a cohort of 97 individuals, consisting of 53 males and 44 females, with an average age of 69.74 years (± 12.24). The age distribution of participants spanned from 33 to 95 years; 58 consecutive COVID-19 and 39 CIP cases. We identified 269 pneumonia lesions, comprising 159 COVID-19-positive lesions and 110 CIP lesions. Table **1** provides a comprehensive overview of the clinical characteristics exhibited by the patients included in both the training and validation cohorts. All lesions were allocated randomly into training and validation cohorts. The Mann-Whitney U test revealed no statistically significant differences in radiomics features between the two cohorts (*P* > 0.05) based on the manually segmented pneumonia images of 10 randomly selected patients.

### 3.2 Feature Extraction and Screening Results

**Fig. (1):**
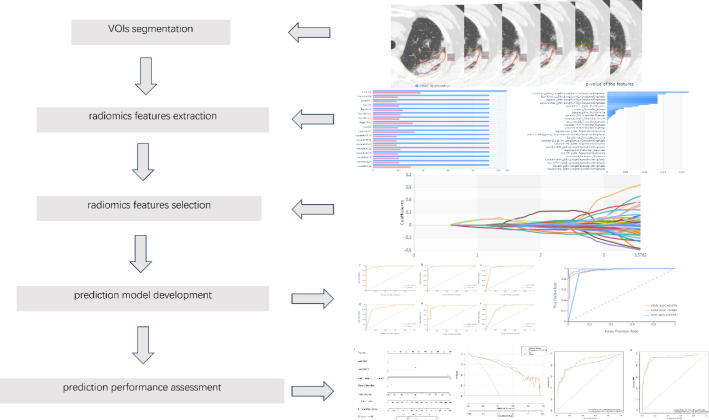
Construction of the workflow model and radiomics analysis. (**a**) segmentation of VOIs, (**b**) the process of extracting radiomic features, (**c**) the procedure for selecting radiomic features, (**d**) the creation of the predictive model utilizing the training dataset, and (**e**) predictive performance assessment.

Initially, utilizing the variance threshold approach, we identified 421 features from an initial pool of 1688. Subsequently, the SelectKBest technique was employed, we narrowed it down to 149 features (Fig. **1**). Finally, we identified 33 optimal features using the LASSO algorithm Fig. (**2a-c**) and Table **2**. The Rad-score demonstrated a statistically significant distinction between patients with CIP and those with COVID-19, with a p-value of less than 0.05, as calculated as described in the Supplementary Data.

### 3.3 Diagnostic Performance of Radiomics Models

**Fig. (3):**
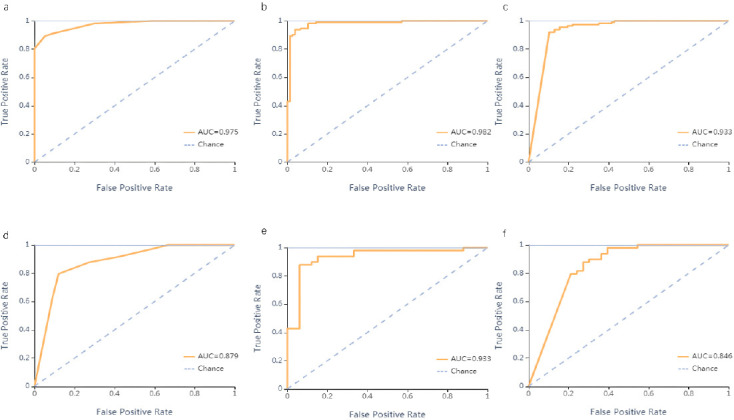
ROC curves of the KNN (**a**), SVM (**b**), and SGD (**c**) classifiers in the training set. ROC curves of the KNN (**d**), SVM (**e**), and SGD (**f**) classifiers in the validation set.

**Fig. (4):**
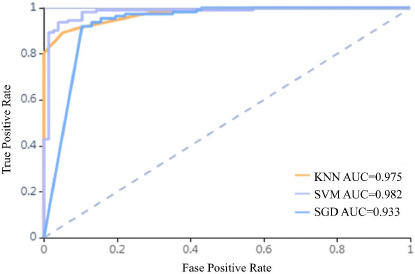
DeLong test results of the KNN, SVM, and SGD classifiers in the training set.

The findings of the ROC curve assessment for the training and validation datasets are displayed in Tables **3** and **4**, respectively. Utilizing an SVM classifier for training, the AUC achieved for the training dataset was 0.988 in distinguishing CIP from COVID-19 (95% confidence interval: 0.960-1.000; sensitivity: 0.94, specificity: 0.97). Conversely, the AUC for the validation dataset was recorded at 0.945 (95% confidence interval: 0.874-1.000; sensitivity: 0.96, specificity: 0.82) (Fig. **3a-f**). The SVM classifier achieved the highest accuracy among the three models tested (Fig. **4**). The four metrics used to evaluate the classifier -precision, recall, F1-score, and support -are thoroughly presented in Tables **3** and **4**.

### 3.4 Construction of Radiomics Nomograms and Evaluation of the Efficacy of Various Models

**Fig. (5a,b):**
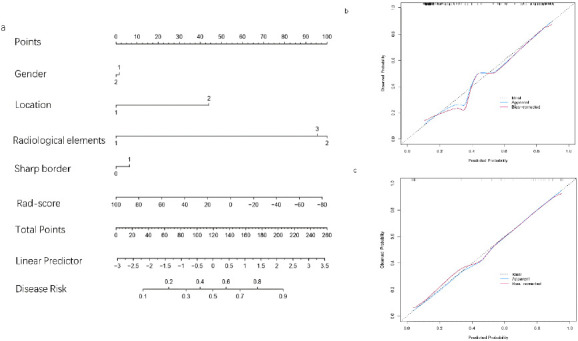
Herein, we present the radiomics nomogram along with its associated calibration curves. This nomogram integrates multiple variables, such as Rad-score, gender, anatomical site, radiological characteristics, and data regarding sharp borders, and was constructed utilizing the training dataset. Calibration curves for this nomogram are shown for both the training and validation datasets. These curves are essential for assessing how well the model fits the data. The dotted line at a 45° angle represents the ideal prediction standard, while the solid line, which has been adjusted for bias, indicates the model's predictive performance. A solid line that closely follows the ideal prediction line suggests that the nomogram has a high level of predictive accuracy.

The Rad-score, along with factors such as gender, geographical location, radiological characteristics, and the presence of sharp borders, was collectively utilized to develop a radiomics nomogram (Fig. **5a**). Calibration curves illustrating the performance of the radiomics nomogram are depicted in Fig. (**5b** and **c**) for both the training and validation datasets. Furthermore, the ROC curves for both the radiomics signature and the nomogram are shown in Fig. (**6a, b**). An analysis of the diagnostic efficacy of each model is presented in Table **5**, with the radiomics nomogram exhibiting a markedly enhanced predictive performance. Importantly, the area under the curve for the radiomics nomogram was significantly greater than that of the clinical factors model in the training cohort (*p* = 0.02638), indicating robust calibration in both training and validation datasets. The DCA results from the training dataset, illustrated in Fig. (**7**), reveal that the radiomics nomogram yields a higher net benefit compared to the clinical model.

## 4 DISCUSSION

In this retrospective analysis, we constructed and validated multiple classification models utilizing CT radiomics to differentiate between CIP and COVID-19. The developed radiomics nomograms exhibited robust efficacy in both the training and validation datasets, with AUC values of 0.853 and 0.932, respectively, affirming the feasibility of the CT-based radiomics nomogram. The results of the decision curve analysis indicated that the radiomics nomogram provides substantial benefits compared to the clinical model in distinguishing between CIP and COVID-19, showcasing the nomogram's robustness and accuracy in classifying pneumonitis subtypes.

CIP and COVID-19 pneumonia are believed to exhibit similar underlying biological processes, including the heightened activation of immune cells and an elevation in pro-inflammatory cytokine levels [25]. Our findings, consistent with prior studies [26, 27], suggest that specific radiological features can aid in differentiating between CIP and COVID-19 pneumonia. While typical radiological findings of CIP and COVID-19 have been reported, their suggestive nature and the broad radiological [28, 29]spectrum of pneumonitis underscore the significance of radiomics in this differentiation.

Recently, the field of radiomics has been utilized to detect nuanced alterations that are not discernible through visual evaluation of CT imaging [30]. Wang et al. employed artificial intelligence algorithms to integrate findings from chest CT scans with clinical manifestations, exposure history, and laboratory examinations for the purpose of diagnosing COVID-19 [31]. Qiu and colleagues developed a radiomics model utilizing deep learning techniques, which is designed to differentiate between viral pneumonia cases caused by COVID-19 and those resulting from other viral infections [16]. Qiu and colleagues created and confirmed the efficacy of a radiomics nomogram that employs computed tomography to differentiate between pneumonitis associated with ICIs and that induced by radiation therapy in patients diagnosed with non-small cell lung cancer. These studies showcase the potential of radiomics in identifying lung inflammation. To the best of our knowledge, this research represents the first attempt to develop and validate a radiomics nomogram that differentiates COVID-19 pneumonia from other types of pneumonia. Our results confirm the application of quantitative radiomic features over qualitative radiological factors in distinguishing between different types of pneumonitis [32]. Recently, radiomics features have been identified that can predict subsequent immunotherapy-related pneumonitis, highlighting small differences in image intensities that are not visible to the unaided eyes [33].

Artificial intelligence (AI), particularly through the use of deep learning methodologies, demonstrates considerable potential in the field of radiological imaging due to its ability to identify and evaluate various features [34]. Various studies have reported on the performance of deep learning frameworks for COVID-19 pneumonia [30]. Recent investigations [35] indicate that deep learning algorithms struggle to distinguish between pneumonia caused by COVID-19 and pneumonitis related to immunotherapy. This finding contrasts with our own results. The similarities observed in chest CT scans across different conditions suggest that various causes can trigger similar lung response mechanisms. As a result, assessments based on the characteristics of lung lesions, including their volume, morphology, or density, may prove inadequate for the development of robust deep learning models. Moreover, although deep learning models typically perform better with large datasets than manual feature extraction [31, 36], large datasets are not always feasible in medicine due to disease rarity, data collection barriers, and other clinical constraints. For smaller datasets, feature engineering might be a more suitable machine learning approach, offering significant benefits for medical imaging analysis through radiomics [37].

The SVM radiomics classifier is widely utilized by researchers to address practical challenges on a broad scale, particularly when resources are limited [38]. The constructed model, along with the subsequent validation outcomes, has demonstrated that the SVM radiomics classifier is a reliable indicator for differentiating CIP from COVID-19 pneumonia in cancer patients, reflecting their distinct pathological foundations. Various categories of pathogens responsible for pneumonia elicit diverse immune responses from lymphocytes and monocytes at the onset of the infection [39], indicating that the radiomic model may function as a diagnostic tool for different pneumonia subtypes. This provides healthcare professionals with additional diagnostic insights and facilitates the advancement of individualized, precision-based treatment.

## 5 STUDY LIMITATIONS

Nonetheless, this research is subject to various limitations. Firstly, as this research constitutes a retrospective analysis, the training and validation datasets were obtained from a single institution; therefore, its conclusions may not be broadly applicable to other research centers. Future research will focus on incorporating samples from diverse institutions for external validation. Secondly, the exclusion of other viral or bacterial pneumonias from this study limits the model's ability to diagnose different types of infectious pneumonia accurately. Additionally, there is a lack of direct comparison between the performances of radiomics and deep learning clinical models. Lastly, the process of manually segmenting three-dimensional regions of interest is often labor-intensive and intricate, especially when dealing with lesions that exhibit ambiguous borders. Future studies should aim to develop automatic segmentation methods that are both reliable and reproducible.

## CONCLUSION

In summary, the broad range of radiologic presentations of CIP and COVID-19 presents significant diagnostic and management challenges in clinical settings. The radiomics nomogram, a noninvasive and quantitative method, has the capability to effectively distinguish between COVID-19-related conditions and CIP, thereby enhancing radiologists' diagnostic capabilities through machine learning predictions. This is particularly valuable when the healthcare system is under strain.

## Figures and Tables

**Fig. (2):**
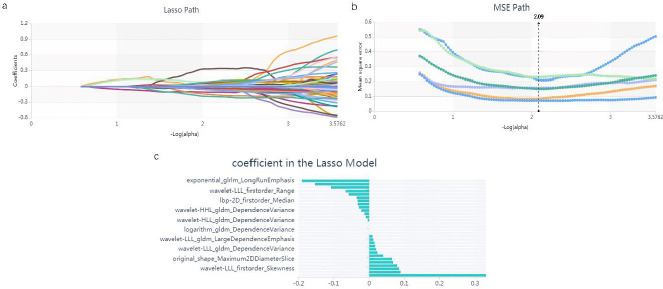
The application of the LASSO algorithm for feature selection is demonstrated through several analyses: (**a**) the LASSO path, (**b**) the Mean Squared Error (MSE) path, and (**c**) the coefficients derived from the LASSO model. Through the implementation of the LASSO model, a total of 30 features were identified, which aligned with the optimal value of alpha.

**Fig. (6):**
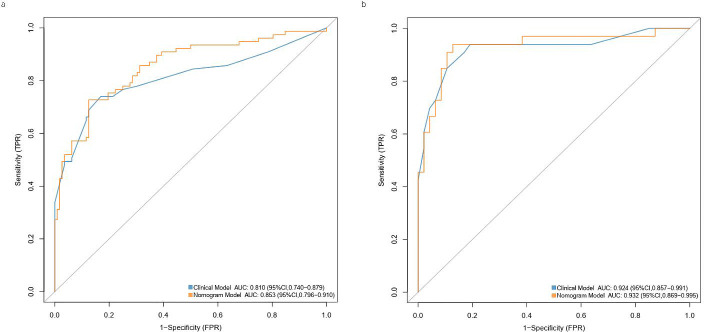
ROC curves of the clinical model and the radiomics nomogram model in the training (**a**) and validation (**b**) sets.

**Fig. (7):**
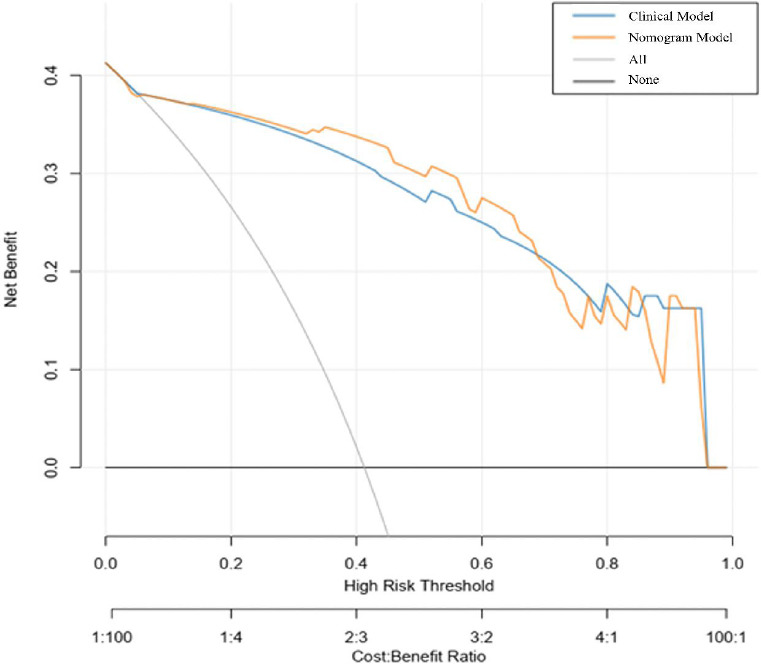
To evaluate the efficacy of the radiomics nomogram in conjunction with clinical variables, a decision curve analysis (DCA) was conducted. In this framework, the X-axis corresponds to the threshold probability, whereas the Y-axis illustrates the net benefit. The yellow line denotes the net benefit associated with the radiomics nomogram, in contrast to the blue line, which signifies the net benefit derived from the clinical model. Notably, the radiomics nomogram demonstrated a superior net benefit when compared to the clinical model.

**Table 1:** The clinical and radiological characteristics of individuals diagnosed with pneumonitis were examined within both the training and validation datasets.

**Property**	**Training Data Set**			**Validation Data Set**		
-	**CIP**	**COVID-19**	***p***	**CIP**	**COVID-19**	***p***
Pneumonia lesion (n)	77	112	-	33	47	-
Sex (female/male)	59/18	63/49	0.004	21/12	24/23	0.264
Age (years)	67.8±8.1	69.4±12.1	0.134	69.8±8.4	68.9±12.8	0.058
Location	-	-	-	-	-	-
Subpleural	44(57.1)	92 (82.1)	<0.001	13 (39.4)	41 (87.2)	<0.001
Peribronchovascular	33(42.9)	20 (17.9)	-	20 (60.6)	6 (12.8)	-
Radiological elements	-	-	-	-	-	-
GGO	24 (31.2)	98 (87.5)	<0.001	9 (27.3)	44 (93.6)	<0.001
Consolidation	31 (40.3)	7 (6.2)	-	10 (30.3)	1 (2.1)	-
GGO + consolidation	22 (28.6)	7 (6.2)	-	14 (42.4)	2 (4.3)	-
Sharp border	-	-	-	-	-	-
Yes	60 (77.9)	57 (50.9)	<0.001	23 (69.7)	14 (29.8)	<0.001
No	17(22.1)	55 (49.1)	-	10 (30.3)	33 (70.2)	-

**Table 2:** LASSO coefficients of 33 radiomics features.

**Radiomics feature**	**Radiomics class**	**Filter**	**Coefficient**
DependenceVariance	gldm	wavelet-LHH	-0.07213
DependenceVariance	gldm	wavelet-LHH	-0.04389
DependenceVariance	gldm	wavelet-LHH	-0.02387
LargeDependenceEmphasis	gldm	exponential	0.13007
LargeDependenceLowGrayLevelEmphasis	gldm	exponential	1.35811
LargeDependenceHighGrayLevelEmphasis	gldm	exponential	5.16929
LargeDependenceEmphasis	gldm	gradient	7.04904
LargeDependenceLowGrayLevelEmphasis	gldm	gradient	7.04904
LargeDependenceHighGrayLevelEmphasis	gldm	gradient	7.04904
LargeDependenceEmphasis	gldm	square	7.04904
LargeDependenceLowGrayLevelEmphasis	gldm	lbp-3D-m1	1.17484
LargeDependenceEmphasis	gldm	wavelet-LLL	0.01653
LargeDependenceLowGrayLevelEmphasis	gldm	wavelet-LLL	0.11568
Skewness	firstorder	squareroot	0.06534
Skewness	firstorder	wavelet-LLL	0.0248
Range	firstorder	wavelet-LLL	-0.08715
LargeDependenceHighGrayLevelEmphasis	gldm	wavelet-LLL	-0.06443
Skewness	firstorder	square	0.0172
Kurtosis	firstorder	wavelet-HLL	-0.01357
Kurtosis	firstorder	lbp-3D-k	0.00301
Kurtosis	firstorder	wavelet-LLL	-0.06192
RunVariance	glrlm	original	-0.00776
RunVariance	glrlm	logarithm	-2.13821
RunVariance	glrlm	squareroot	-1.17484
Kurtosis	firstorder	wavelet-LHH	0.00793
LargeDependenceLowGrayLevelEmphasis	gldm	lbp-3D-k	0.01318
LongRunEmphasis	gldm	exponential	-0.00851
LongRunHighGrayLevelEmphasis	gldm	exponential	-3.05458
GrayLevelNonUniformity	gldm	wavelet-LHH	-0.07509
RunVariance	gldm	lbp-3D-k	0.01558
LargeAreaLowGrayLevelEmphasis	gldm	wavelet-HLH	-0.01763
RunLengthNonUniformity	gldm	exponential	-0.00148
RunLengthNonUniformity	gldm	gradient	-1.38631

**Table 3:** Results of radiomics analysis for classifications of the training cohort.

**Classifiers**	**AUC**	**95% CI**	**Accuracy**	**Sensitivity**	**Specificity**	**F1-score**
KNN	0.975	0.933 - 1.000	0.940	0.910	0.890	0.920
SVM	0.982	0.945 - 1.000	0.950	0.940	0.920	0.950
SGD	0.933	0.890 - 0.976	0.930	0.920	0.900	0.920

**Abbreviations:** AUC, area under the curve; KNN, K-nearest neighbor; SVM, support vector machine; SGD, stochastic gradient descent.

**Table 4:** Results of radiomics analysis for classifications of the validation cohort.

**Classifiers**	**AUC**	**95% CI**	**Accuracy**	**Sensitivity**	**Specificity**	**F1-score**
KNN	0.879	0.788-0.970	0.830	0.880	0.730	0.850
SVM	0.933	0.861-1.000	0.900	0.940	0.850	0.920
SGD	0.846	0.754-0.938	0.850	0.800	0.790	0.820

**Abbreviations:**AUC, area under the curve; KNN, k-nearest neighbor; SVM, support vector machine; SGD, stochastic gradient descent.

**Table 5:** The effectiveness of the clinical model and the nomogram model in diagnostic applications.

**-**	**Training Data Set**		**Validation Data Set**	
	**Clinical Model**	**Nomogram Model**	**Clinical Model**	**Nomogram Model**
Cutoff	0.368	0.499	0.269	0.464
C-index	0.810	0.855	0.923	0.932
AUC (95% CI)	0.810 (0.740-0.879)	0.853 (0.796-0.910)	0.924 (0.857-0.991)	0.932 (0.869-.996)
Sensitivity	0.740	0.727	0.939	0.939
Specificity	0.830	0.875	0.809	0.872
Accuracy	0.794	0.815	0.863	0.900
PPV	0.750	0.800	0.775	0.838
NPV	0.823	0.824	0.950	0.953

**Abbreviations:** AUC, area under the curve; NPV, negative predictive value; PPV, positive predictive value.

## Data Availability

The datasets used or analyzed during the current study are available from the corresponding author [Z.Z] on reasonable request.

## LIST OF ABBREVIATIONS

ANOVA: Analysis of variance
AUC: Area under the curve
CI: Confidence interval
CIP: Checkpoint inhibitor-related pneumonitis
COVID-19: Coronavirus Disease 2019
DCA: Decision curve analysis
GLCM: Gray-level co-occurrence matrix
GLRLM: Gray-level run length matrix
GLSZM: Gray-level size zone matrix
ICC: Inter-/intra-class correlation coefficient
ICI: Immune checkpoint inhibitor
KNN: k-nearest neighbor
LASSO: Least absolute shrinkage and selection operator
Rad-score: Radiomics score
ROC: Receiver operating characteristic
ROI: Region of interest
VOI: Volume of interest
SGD: Stochastic gradient descent
SVM: Support vector machine

## STANDARDS OF REPORTING

STROBE guidelines were followed.
