# Supplementary material for: Differentiating Immune Checkpoint Inhibitor-Related Pneumonitis from COVID-19 Pneumonia Using a CT-based Radiomics Nomogram
Source: Curr Med Imaging. 2025 Oct 21;21:e15734056399950. doi: 10.2174/0115734056399950251003114150 (PMC13126303; doi:10.2174/0115734056399950251003114150)
Supplement: Supplementary file 1 — Supplementary material is available on the publisher’s website along with the published article. [file CMIM-21-E15734056399950_SD1.pdf]

1573-4056/25 *Send Orders for Reprints to reprints@benthamscience.net*

BENTHAM SCIENCE

CrossMark

Current Medical Imaging

Content list available at: https://benthamscience.com/journals/cmir

Supplementary Material

Differentiating Immune Checkpoint Inhibitor-Related Pneumonitis from COVID-19 Pneumonia Using a CT-based Radiomics Nomogram

Fengfeng Yang[1,#], Zhengyang Li[1,#,*], Di Yin[1], Yang Jing[2] and Yang Zhao[1,*]

[1]*Department of Radiology, The Second Hospital of Tianjin Medical University, Tianjin 300211, China*
[2]*Huiying Medical Technology Co., Ltd., Beijing 100192, China*

Supplementary data.

The Rad-score was calculated using the following formula:
Rad-score= -0.07213×wavelet-LHH_gldm_DependenceVariance -0.04389
×wavelet-HLH_gldm_DependenceVariance-0.02387
×wavelet-HHL_gldm_DependenceVariance+0.13007
×exponential_gldm_ LargeDependenceEmphasis+1.35811
×exponential_gldm_ LargeDependenceLowGrayLevelEmphasis+5.16929
×exponential_gldm_ LargeDependenceHighGrayLevelEmphasis+7.04904
×gradient_gldm_LargeDependenceEmphasis+7.04904
×gradient_gldm_LargeDependenceLowGrayLevelEmphasis+7.04904
×gradient_gldm_LargeDependenceHighGrayLevelEmphasis+7.04904
×square_gldm_LargeDependenceEmphasis+1.17484
×lbp-3D-m1_gldm_LargeDependenceLowGrayLevelEmphasis+0.01653
×wavelet-LLL_gldm_LargeDependenceEmphasis+0.11568
×wavelet-HHH_gldm_LargeDependenceLowGrayLevelEmphasis+0.06534
×squareroot_firstorder_Skewness+0.02480
×wavelet-LLL_firstorder_Skewness-0.08715
×wavelet-HLL_firstorder_Range-0.06443
×wavelet-LLH_gldm_LargeDependenceHighGrayLevelEmphasis+0.01720
×square_firstorder_Skewness-0.01357
×wavelet-HLL_firstorder_Kurtosis+0.00301
×lbp-3D-k_firstorder_Kurtosis-0.06192
×wavelet-LLL_firstorder_Kurtosis-0.00776
×original_glrlm_RunVariance-2.13821
×logarithm_glrlm_RunVariance-1.17484
×squareroot_glrlm_RunVariance+0.00793
×wavelet-LHH_firstorder_Kurtosis+0.01318
×lbp-3D-k_gldm_LargeDependenceLowGrayLevelEmphasis-0.00851
×exponential_glrlm_LongRunEmphasis-3.05458
×exponential_glrlm_LongRunHighGrayLevelEmphasis-0.07509

DOI: 10.2174/0115734056399950251003114150, 2025, *21*, e15734056399950

×wavelet-LHH_glszm_GrayLevelNonUniformity+0.01558
×lbp-3D-k_glrlm_RunVariance-0.01763
×wavelet-HLH_glszm_LargeAreaLowGrayLevelEmphasis-0.00148
×exponential_glrlm_RunLengthNonUniformity-1.38631
×gradient_glrlm_RunLengthNonUniformity

© 2025 The Author(s). Published by Bentham Science Publisher.

This is an open access article distributed under the terms of the Creative Commons Attribution 4.0 International Public License (CC-BY 4.0), a copy of which is available at: https://creativecommons.org/licenses/by/4.0/legalcode. This license permits unrestricted use, distribution, and reproduction in any medium, provided the original author and source are credited.
